# Short Reports of Twelve Cases of Ligature of Some of the Main Arteries Occurring during the Past Few Months at the Bristol Royal Infirmary

**Published:** 1884-06

**Authors:** J. Paul Bush

**Affiliations:** House Surgeon


### Clinical Records.

---

SHORT REPORTS OF TWELVE CASES OF LIGATURE OF SOME OF THE MAIN ARTERIES occurring during the past few months at the Bristol Royal Infirmary. By J. PAUL BUSH, House Surgeon.

LIGATURE OF THE COMMON CAROTID.

W. J., æt. 65, collier. Under Mr. Dowson. Admitted for a hard tumour occupying the left submaxillary region. Patient had noticed it for four years; it had been gradually increasing in size ever since, causing much pain for last twelve months. On admission tumour was the size of a small hen's egg, and was evidently in close relation with the vessels of the neck, and was not attached to the jaw.

Ten days after admission the tumour was removed by the knife (and found on microscopic examination to be a sarcoma); severe hæmorrhage followed, from the divided vessels running into the growth, which necessitated ligature of the common carotid (above the omo-hyoid muscle, with a catgut ligature and the usual antiseptic precautions); the hæmorrhage then ceased; the patient made a rapid recovery, the wound was healed in three weeks, and the patient allowed up.

Five weeks after the operation acute panophthalmitis of the left eye set in, and the temperature rose to 101° without any previous symptoms; during the next five days the eye became completely disorganized; on the forty-second day after ligature of the carotid the patient suddenly lost the power of motion in the right arm (only), and at the same time became aphasic. Consciousness was considerably impaired, and there were involuntary evacuations; the temperature at this time ranging from 104°—105°. The patient remained much in this condition for four weeks, but was perhaps becoming rather more conscious, and had some slight control over his evacuations, when he was removed by his friends (ten weeks after ligature of the common carotid).

He died about one week after being taken away, his friends say quite suddenly.

### LIGATURE OF THE COMMON CAROTID.

W. T., æt. 52. Under Mr. Prichard. Admitted for tumour at back of left orbit; this had been noticed for five months. On admission sight was completely gone in the left eye, the globe was protruding, and over the left temporal region was a soft swelling. One week after admission the eye was removed and an incision made over the temporal region, when a large mass of soft tissue was found (this proved to be round-celled sarcoma under the microscope); this growth appeared to be continuous with the mass at the back of the orbit; an attempt was made to remove it. There was severe hæmorrhage; pressure, the cautery and perchloride of iron were applied; oozing continued for twenty-four hours, when the common carotid was tied with a thick catgut ligature above the omo-hyoid muscle. The hæmorrhage ceased, but the patient died eight hours after the operation; the coma which came on immediately after the first operation steadily increasing up to the time of death. The *post mortem* examination showed the whole of left orbital and temporal regions invaded with sarcoma.

### LIGATURE OF THE COMMON CAROTID.

C. V., æt. 63. Under Mr. Cross. Patient admitted for epithelioma of the orbital region. Eight years ago a small ulcer appeared on the lower lid. This was removed; the ulceration soon recurred, and rapidly spread to the globe of the eye, nose and cheek.

On admission the whole of the upper half of the left side of the face was replaced by a deep excavation; this had implicated the whole of the orbit, had spread into the nose and nearly down to the angle of the mouth and back to the ear, exposing the bones. Patient had had great pain, which occurred at intervals, and was always relieved by the severe hæmorrhage which for the past three or four months had come on every two or three days.

Four weeks after admission, during which time the pain and hæmorrhage increased in spite of various local applications, the left common carotid was tied at the omo-hyoid muscle with a thick catgut ligature; antiseptics, horse-hair drainage. The temperature never rose above 99°, and there were no cerebral symptoms whatever; and in eight days the wound was healed and the sutures removed; and in three weeks the patient was allowed to sit up, and was sent home at the end of the fourth week.

The ligature of the common carotid seems to have had little or no effect either on the progress of the growth or on the hæmorrhage. About two weeks after going out the hæmorrhage and pain steadily increased, but the patient was able to walk about, and he retained all his faculties up to the day of his death, four months after the operation.

LIGATURE OF THE COMMON CAROTID.

H. H., æt. 68. Under Mr. Cross. Six months before admission patient noticed a swelling in the right side of the forehead, and for some weeks before this she had had severe pain in the right eye and side of face. Patient had been getting about up to a week before admission, when her present condition came on.

On admission patient was semi-conscious, occasionally complaining of pain in the head; there was partial hemiplegia of the left side of the body; implicating the right temporal and right half of the frontal bone, and extending to below the *zygoma*, was a soft tumour pulsating synchronously with the temporal artery; no bruit could be heard; pressure on the temporal or carotid did not diminish the size of the tumour, but the pulsation was lessened, but did not cease entirely; the right eye was pushed considerably forwards, the conjunctiva non-sensitive; both pupils acted very slowly to light. The day after admission, as the coma was increasing, the common carotid was tied antiseptically with a catgut ligature above the omo-hyoid muscle; the tumour was very slightly diminished in size, but the coma, &c. did not alter. Two days after the operation the unconsciousness increased slowly, and the patient died five days after the operation.

*Post mortem* examination: The tumour was found to be a small round-celled sarcoma of the skull, pressing on the brain but not invading the dura mater; there were numerous large vessels running directly into the growth.

FEMORAL ANEURISM. LIGATURE OF FEMORAL.

T. T., æt. 31, labourer. Under Mr. Board. Fifteen months before admission patient first felt discomfort in the lower and inner part of the right thigh, and about two months ago he found a small swelling in this position, which had steadily increased to its present size. On admission he had an ovoid swelling in the lower quarter of the right thigh; over its posterior and inner aspect this swelling was $4\frac{1}{2}$ inches long and $3\frac{3}{4}$ inches broad; it pulsated visibly; this pulsation was completely controlled by pressure on the femoral above, but not by flexion of the knee or thigh; there was a double bruit to be heard over the swelling and up the femoral; veins were much enlarged; there was no œdema of the limb. Posterior tibial could be felt pulsating less distinctly than in the left leg; there was no evidence of arterial disease elsewhere; no history of syphilis or rheumatism; but the patient had been a hard drinker all his life.

One week after admission the aneurism was increasing in size, and the patient was in more pain.

Digital compression of femoral for six hours; no result; temperature rose to 102° and continued high for three days; compression was then again applied for four hours, with no result. One week after this the femoral was ligatured in Scarpa's triangle with a chromicised catgut ligature, and with the usual antiseptic precautions; the pulsation ceased entirely. The wound healed rapidly, and the patient was up in one month, and went out soon after this. Patient was seen two weeks afterwards, when he could use his leg fairly well.

Patient was re-admitted two years after this, with the following history: Remained well till six weeks ago, since which time he has had severe pains, in the right leg and foot, which has ever since he went out been much colder than the left leg. Two days before admission was quite suddenly seized with very severe pain in the calf of right leg. The right foot on admission was blue and cold, and over the toes were several bullæ; the pain was steadily increasing, and was completely confined to the portion of skin supplied by the internal saphenous nerve; over this area there was also diminished perception of localization. In the course of two weeks the skin over the toes dried up and peeled off, leaving a few points of extravasation; these soon were absorbed, and the toes then slowly regained their normal colour, but never their proper temperature, neither was sensation, properly re-established. The patient was allowed up, and the condition of the foot did not alter; he went home seven weeks after admission, being able to walk well without pain.

ANEURISM OF FEMORAL. LIGATURE OF FEMORAL.

J. C., æt. 33, hawker. Under Mr. Board. Patient admitted for a pulsating tumour in the lower third of thigh; noticed for the first time only three weeks before admission. There was a distinct history of a hard sore, followed by all the secondary symptoms, and tertiary ulcerations and necrosis of tibiæ.

On admission patient was in a very low state, and covered with rupia. In the lower third of Hunter's canal was an elastic swelling the size of a hen's egg, which had an expansile pulsation and bruit, which ceased on pressure applied to the femoral. After three weeks' treatment patient was gradually getting worse, and the aneurism was increasing in size; the femoral was exposed at the apex of Scarpa's triangle, and tied antiseptically with one of Barwell's flat ox-aorta ligatures. Venous hæmorrhage occurred at the time of the operation, due to a wound of the vein, but this ceased entirely when pressure was applied. The day after the operation no pulsation could be made out, and the aneurism was greatly diminished in size; limb, warm, and a good colour; patient says he is in much less pain.

On the second day the patient's general condition was not so good, and he gradually sank, and died on the fifth day after ligature was applied. *Post mortem:* The aneurism was found filled with soft fibrine, and there was very extensive disease of all the internal organs. The spleen was enormously enlarged.

### ANEURISM OF POPLITEAL. LIGATURE OF FEMORAL.

R. B., æt. 32, miner. Under Mr. Greig Smith. Admitted for a pulsating swelling in right popliteal space. This had only been noticed for three weeks, and patient had only been for one week off work on account of the pain in knee. On admission right knee was slightly flexed, and could not be extended without much pain; there was slight effusion into joint, with marked œdema and dilated veins of leg and ankle. The anterior and posterior tibial arteries could be felt pulsating less plainly than in opposite leg. Filling up the whole of the popliteal space was a large elastic tumour, with expansile pulsation (stopped by compression of the femoral), and very distinct thrill and loud rasping murmur. One week after admission there was no improvement with the rest, &c. Digital compression was applied on two occasions, in all for eighteen hours, the only effect being a slight temporary hardening of the aneurism.

Three weeks after admission the femoral was ligatured at the apex of Scarpa's triangle with a catgut ligature and the usual antiseptic precautions. Pulsation immediately ceased, but was again noticed slightly on the second day. At the end of the third day no pulsation could be made out. The wound healed by first intension without any complications. In ten days there only remained some slight thickening round the original seat of the aneurism. Three weeks after the operation the patient was up, and ten days after this went out cured. Reported himself, six months after going out, as quite cured and doing his ordinary work.

### POPLITEAL ANEURISM. LIGATURE OF THE FEMORAL.

C. C., æt. 44, ex-soldier. Under Mr. Cross. Patient had served in the Hussars all his life. History of syphilis twenty years ago. History of pain and stiffness in knee for three months. Patient noticed the swelling one month before admission, when he was doing a good deal of extra work in the saddle. On admission, in the upper portion of the popliteal space there was a hard swelling the size of a cricket-ball, with expansile pulsation, thrill and rough bruit, which ceased on compressing the femoral; the knee was much flexed and stiff; the foot and leg were œdematous, and there was much pain in the leg and toes; the patient was in the habit of taking morphia frequently; there was a mitral systolic bruit, and the brachial arteries were hard and tortuous. Two days after admission Esmarch's bandage was applied without result, followed by compression by a shot-bag placed over the femoral in Scarpa's triangle for thirty-six hours, also with no result. After two weeks' rest with splint, &c., the aneurism was still rapidly increasing in size, the skin over it was dusky, and the œdema and venous congestion of the foot were increasing. The femoral was ligatured antiseptically at the apex of Scarpa's triangle with a catgut ligature; no drainage. Next day there was slight pulsation noticed in the aneurism, but none after this. Ten days after the operation the aneurism was soft and flaccid, but no pulsation; the foot still remained cold and œdematous, but the patient's general condition was much improved. Four weeks after the operation the aneurismal sac showed no tendency to contract. An antiseptic incision was made into the tumour, which was found to be full of recent black blood-clot, surrounded by a small portion of laminated fibrine not completely decolourised; the whole of this was turned out and the cavity drained. Four weeks after this a patch of gangrene showed on the little toe; the tumour was then much smaller, but still discharging slightly. Three weeks after this the wound was completely healed, and there was only some slight thickening left in the popliteal space, the knee being flexed almost to a right angle. An attempt was made to straighten the limb by a Macintyre splint; this increased the slough on the little toe, and some superficial ulceration of the second toe and outer side of the foot followed.

Patient went out three months and a half after admission, and was seen some months afterwards, when he had lost the two toes, but was walking about with a bent and stiff knee.

### TRAUMATIC ANEURISM OF RADIAL. LIGATURE OF BRACHIAL.

W. S., æt. 38, sugar boiler. Under Mr. Prichard. On admission patient was suffering from a punctured wound at the centre of the bend of the right elbow. A probe could be passed in three or four inches, and the point could be felt on the outer side of the head of the radius, immediately beneath the skin. There had evidently been severe hæmorrhage before admission, and there was a good deal of extravasated blood in front of the elbow-joint. Both radial and ulnar arteries could be felt pulsating slightly. The limb was put on a splint, and no further hæmorrhage occurred. On the third day after admission the temperature had risen to 101°; the arm was swollen and very painful; the swelling in front of the elbow was much increased and pulsating; the radial and ulnar arteries could not be felt. A free incision was made over the mass of extravasated blood, but the bleeding point (? in the upper tenth of the radial, as the blood was seen *oozing* freely up from beneath the supinator longus) could not be found. The brachial was ligatured with a catgut ligature immediately above its bifurcation, and the hæmorrhage ceased. The temperature was normal by the third day, and the wound healed slowly by granulation; and the patient went out cured in eight weeks. Patient showed himself one month after this, when there was no stiffness of joint, and no pain or coldness of the arm or hand.

### TRAUMATIC ANEURISM AND LIGATURE OF RADIAL.

P. R., æt. 19. Under Mr. Prichard. History was, five weeks before admission the patient sustained a punctured wound at the junction of the lower and middle third of the right forearm. There was profuse hæmorrhage at the time of the accident; no vessel was tied, the wound was sewn up, and no bleeding occurred again; the wound healed by first intention, and the patient was working four days after his accident. Three weeks after this the patient noticed a slight swelling round the seat of the original injury; since this the swelling has steadily increased in size up to the time of admission.

On admission there was a tumour the size of a pigeon's egg, situated on the anterior radial aspect at the junction of the middle and lower third of the right forearm, presenting the usual physical signs of an aneurism. On compressing the radial above, the tumour became soft, and could be emptied, but refilled slowly unless pressure was also applied to the ulnar.

One week after admission the tumour was considerably larger. The radial was ligatured antiseptically with a catgut ligature above and below the aneurism. (On drawing the upper ligature tight pulsation continued till a ligature was also applied below.) The wounds healed by granulation. One month after the operation the tumour was still present, though now as a hard, solid mass the size of a cob nut, and gave rise to some pain. An incision was made into it, and a firm blood-clot turned out. The wound healed in ten days, and the patient went out cured. The patient has since been seen, and was then quite cured, and doing the hard manual work of a blacksmith without any further trouble.

### TRAUMATIC ANEURISM OF TEMPORAL.

W. H., æt. 6. Under Mr. Greig Smith. Patient was run over by a heavy cart, and the whole of the upper portion of the scalp was torn off; there was no hæmorrhage at the time of the accident.

Two weeks after the injury an aneurism, the size of a small cob nut, was noticed in the course of the left temporal, about half an inch above the external auditory meatus. Three days after this, during a fit of coughing, the aneurism ruptured, and there was severe hæmorrhage; the wound was enlarged and the two ends of the vessel found, the clot turned out, and a catgut ligature applied above and below the seat of the aneurism; hæmorrhage ceased at once, and the wound healed rapidly. Some necrosis of the skull followed the original injury.

### TRAUMATIC ANEURISM OF TEMPORAL.

A. H., æt. 12. Under Mr. Greig Smith. One month before admission patient sustained a contused wound of the scalp. Since the injury there has been a tumour steadily increasing in size, which has bled freely every few days since injury.

On admission, situated above the right ear and in the course of the posterior temporal artery is a mass of vascular tissue, pulsating, the size of a large hazel-nut. This tumour was removed by the knife, and a large artery running into this was secured by a hare-lip pin. Wound healed in a week. No return of the trouble.

